# RNA-Seq Revealed the Effects of Cold Stress on Different Brain Regions of *Leiocassis longirostris*

**DOI:** 10.3390/ani15142107

**Published:** 2025-07-16

**Authors:** Senyue Liu, Qiang Li, Yongqiang Deng, Zhongwei Wang, Yang Feng, Zhongmeng Zhao, Han Zhao, Lu Zhang, Yuanliang Duan, Zhipeng Huang, Jian Zhou, Chengyan Mou

**Affiliations:** 1 Fisheries Research Institute, Sichuan Academy of Agricultural Sciences (Sichuan Fisheries Research Institute), Chengdu 611731, China; liusenyue@scsaas.cn (S.L.); liq7920@126.com (Q.L.); dyqhxl@126.com (Y.D.); fyang@scsaas.cn (Y.F.); 18227552594@163.com (Z.Z.); zhaohan232323@163.com (H.Z.); zhanglu425@163.com (L.Z.); ylduan@scsaas.cn (Y.D.); caihaiyili12345@163.com (Z.H.); 2 Institute of Hydrobiology, Chinese Academy of Sciences, Wuhan 430072, China; wzw0909@ihb.ac.cn

**Keywords:** cold stress, brain, *Leiocassis longirostris*, rhythm, immunity, transcription

## Abstract

Cold shock represents a prevalent but harmful environmental stress factor that poses significant threats to fish survival and reproductive success. In fish, the brain acts as a central regulator of thermoregulatory processes. Nevertheless, how different brain regions respond molecularly to cold exposure remains largely unknown. To address this, this study systematically investigated the effects of acute cold stress on five specific brain regions of *Leiocassis longirostris* using RNA-seq. The findings demonstrated that all five brain regions were significantly impacted by cold treatment, with the mesencephalon (MB) showing the most substantial changes. GO and KEGG enrichment analyses indicated that cold stress disrupted processes including gene expression regulation, circadian rhythms, and immune function within brain tissues. Through Weighted Gene Co-Expression Network Analysis (WGCNA), the MB was identified as the core responsive region, and the brain’s reaction to cold stress was strongly correlated with circadian rhythm, spliceosome, and ubiquitination. In summary, our investigation demonstrates that the MB represents a principal region for cold stress response in *L. longirostris*, involving alterations in circadian clocks, immune function, and inflammatory responses, alongside suppression of gene expression processes and ubiquitination-mediated proteolysis.

## Simple Summary

The endangered fish *Leiocassis longirostris* suffers is highly susceptible to cold stress during seasonal changes or cold waves, often leading to mass mortality. While the brain regulates thermoregulation, region-specific molecular responses to cold remain poorly understood. This study employed RNA-seq to systematically analyze acute cold stress (4 °C, 24 h) effects across five brain regions in *L. longirostris*. Results revealed that different brain regions have distinct regional responses. Crucially, the mesencephalon (MB) exhibited the most significant changes and was identified as the core responsive region. Cold stress triggered MB-specific circadian rhythms, spliceosome, and ubiquitin-mediated proteolytic changes to coordinate adaptation to environmental stress.

## 1. Introduction

Temperature constitutes one of the pivotal environmental parameters that exert a profound influence on the entire life cycle of fish, and fluctuations in temperature affect their development, growth, reproduction, and metabolism, as well as geographical distribution [1]. In addition, freshwater ecosystems are particularly vulnerable to the effects of global change. At present, cold shock has become a prevalent and deleterious environmental stressor in fish [2]. Fish may encounter exposure to low temperature in their habitats that exceed the adaptive thresholds, posing a significant challenge to their survival and reproduction [3]. Consequently, the capacity to cope with cold stress is of utmost importance for the survival of fish under natural conditions, and delving into the mechanisms by which fish respond to cold stress holds great scientific significance.

The brain is an extremely complex central organ, which can be anatomically divided into five regions [4]: olfactory bulb (OB); pituitary, hypothalamus and forebrain, (FB); mesencephalon (MB); cerebellum (CB); and spinal cord (SC) (Graphical abstract). These regions regulate thermal homeostasis through division of labor and coordination, with responses involving the regulation of neuroendocrine, immune, and other physiological systems [5]. However, the region-specific regulatory networks of each brain region under cold stress remain unclear.

Previous studies have confirmed that the fish brain exhibits significant responses to cold stress. For instance, in female zebrafish (*Danio rerio*), cold stress can induce the downregulation of circadian rhythm regulatory functions in brain tissues, thereby inhibiting the spawning process [6]. Acute cold stress significantly upregulates the expression of genes related to ubiquitination/deubiquitination in the brain tissues of the tropical stenothermal fish tiger barb (*Puntius tetrazona*) [7]. Additionally, transcriptome analysis of the whole brain in *Cyprinus carpio* L. has revealed that RNA splicing and spliceosome-related pathways are significantly enriched after cold stress [8]. However, most of the aforementioned studies have taken the whole brain as the research object and have not yet conducted a systematic analysis of the heterogeneous responses of different brain regions under cold stress. Therefore, exploring the responses of different brain regions to cold stress will not only facilitate in-depth analysis of the interactions and cooperative operation modes among brain regions but also provide important data support for constructing a more comprehensive map of fish brain functions.

*Leiocassis longirostris* is an endangered species listed by the International Union for Conservation of Nature and one of the most economically valuable fish in China [9]. During extreme weather events, such as seasonal transitions and cold wave occurrences, it exhibits a pronounced susceptibility to cold stress, frequently culminating in substantial mortality rates [10]. Although numerous studies have explored the histological and physiological effects of cold acclimation on *L. longirostris* [11], there is limited information on the response of *L. longirostris* to cold stress at transcriptional level. Therefore, this study intends to employ RNA-seq and Weighted Gene Co-Expression Network Analysis (WGCNA) approaches to systematically investigate the response mechanisms to cold stress across different brain regions (OB, FB, MB, CB, SC) in *L. longirostris*, aiming to elucidate region-specific response patterns within the fish brain under cold stress.

## 2. Materials and Methods

### 2.1. Experimental Fish

As previously described [11,12], the study utilized the one-year-old *L. longirostris* obtained from the Sichuan Fisheries Research Institute. Fish displaying absence of injury, strong vitality, and normal swimming behavior were chosen as experimental subjects. These individuals underwent a one-week acclimatization period prior to experimentation. During this temporary rearing phase, environmental conditions were strictly controlled: water temperature was maintained at (26 ± 0.5) °C, dissolved oxygen levels were kept above 6.5 mg·L^−1^, and pH was stabilized at 7.4 ± 0.14. One-third of the water volume was exchanged daily, and the fish were fed twice per day (9 a.m. and 9 p.m.).

Although *L. longirostris* exhibits an optimum growth temperature range of 25 to 28 °C [13], it is notably vulnerable to low temperature stress in winter seasons or under extreme weather conditions, particularly when the cold wave comes. Therefore, fish in this study were divided into two treatments: a control group (26 °C, group C) and a cold stress group (4 °C, group D). Each treatment comprised three biological replicates, with 10 fish per replicate, and the stress time was 24 h [14].

### 2.2. Tissue Sampling

Upon completion of the 24 h-stress period, the brain was divided into five regions and sampled according to the method described by Wang et al. [4] (Graphical abstract). Brain samples were collected immediately (a total of 30 samples, three replicates per brain region for both group C and group D) and stored at −80 °C for subsequent analyses.

### 2.3. RNA Extraction, Library Construction, and Sequencing

Three fish were randomly sampled from both the cold stress (D) and control (C) groups. Total RNA was isolated from all 30 brain tissue samples (three replicates per brain region per treatment group) using Trizol reagent (Takara Bio, Kyoto, Japan), adhering strictly to the manufacturer’s protocol. RNA integrity and purity were assessed via 1% agarose gel electrophoresis and quantified using a Nanodrop 2000 spectrophotometer (Thermo Fisher Scientific, Waltham, MA, USA). Qualified total RNA samples were then used to construct sequencing libraries employing the Illumina Truseq™ RNA Sample Prep Kit (Illumina, San Diego, CA, USA).

Library preparation followed stringent quality criteria: total RNA ≥ 1 ug, RNA concentration ≥ 45 ng·μL^−1^, 1.8 ≤ OD260/280 ≤ 2.2, and 2.0 ≤ OD260/230 ≤ 2.2. Libraries meeting these specifications were subjected to RNA-seq on the Illumina NovaSeq6000 platform by Omicsmart (Guangzhou, China). The RNA-seq data is available in the National Center of Biotechnology Information (NCBI) database via accession number PRJNA1208664.

### 2.4. Transcriptomics (RNA-Seq) Analysis

#### 2.4.1. Sequence Read Processing and Alignment

Raw sequencing reads underwent quality control using Fastp v1.0.1 software to generate high-quality, clean reads. These clean reads were subsequently aligned to the designated reference genome (GDR21070358-2_std_1) using the HISAT2 v2.2.1 software (https://github.com/DaehwanKimLab/hisat2 (accessed on 1 November 2024)) to produce mapped reads for downstream analysis. Transcript assembly from the mapped reads was performed using StringTie v1.3.1 in a reference-based approach (http://ccb.jhu.edu/software/stringtie/index.shtml (accessed on 1 November 2024).

#### 2.4.2. Functional Annotation and Classification

Functional annotation of all assembled transcripts and their corresponding genes was carried out by querying the Gene Ontology (GO) and Kyoto Encyclopedia of Genes and Genomes (KEGG) databases, facilitated by the enrich R v3.4 software package.

#### 2.4.3. Analysis of Differentially Expressed Genes (DEGs) and Functional Enrichment

Transcript read counts were calculated for each sample using RSEM. Normalization of read counts across samples was performed, and differential gene expression (DGE) analysis between the paired cold-stressed and control tissues for each brain region was conducted using the DESeq2 R program (C-OB vs. D-OB, C-FB vs. D-FB, C-MB vs. D-MB, C-CB vs. D-CB, and C-SC vs. D-SC). To control the false discovery rate (FDR), *p*-values were adjusted using the Benjamini-Hochberg approach [15]. Genes exhibiting a |log2 FC| > 1 and adjusted *p* < 0.05 were defined as significantly DEGs.

### 2.5. Weighted Gene Co-Expression Network Analysis (WGCNA)

Gene co-expression network module was identified using the WGCNA algorithm with the corresponding R package (edgeR, version = 3.36.0). The analysis commenced by calculating a gene similarity matrix based on pairwise Pearson correlation coefficients. Following the scale-free network principle, an optimal soft-thresholding power was selected to construct an adjacency matrix, which was subsequently transformed into a topological overlap matrix (TOM). Modules were detected via dynamic tree cutting, using 1-TOM as the distance metric for hierarchical clustering. Modules exhibiting significant correlation (Pearson correlation) with the expression level of the cold acclimation marker gene CIRBP (cold-induced RNA-binding protein) [16,17] were identified as significant modules. Finally, key regulatory networks within significant modules were visualized using Cytoscape v 3.10.3 software.

## 3. Results

### 3.1. Transcriptome Sequencing Data Quality Assessment

Using the Illumina NovaSeq6000 platform, transcriptome sequencing was performed on a total of 30 samples (both group C and group D, with three replicates per brain region). After rigorous quality control and removal of contaminated sequences, high-quality clean reads were generated. These filtered reads were subsequently aligned to the *L. longirostris* reference genome. The sequencing data quality are presented in Table S1, and the outcomes of mapping comparison with reference genomes are depicted in Table S2.

### 3.2. Differential Gene Expression Analysis in Brain Tissue

**Figure 1:**
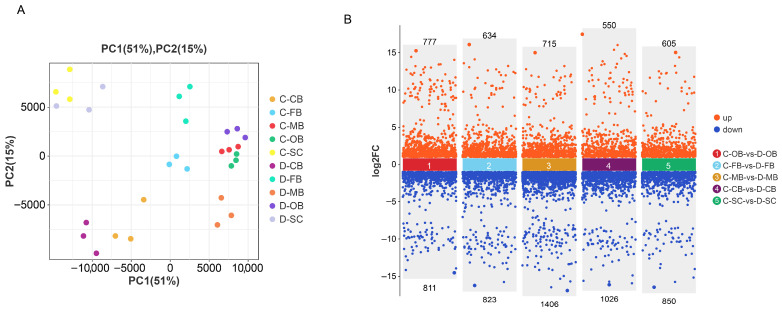
Analysis of DEGs in different brain regions of *L. longirostris* after cold stress. (**A**) Principal component analysis (PCA) of the brain transcriptome. (**B**) The volcano plot shows the distribution of DEGs between each of the two groups (C-OB vs. D-OB, C-FB vs. D-FB, C-MB vs. D-MB, C-CB vs. D-CB and C-SC vs. D-SC).

Following 24 h of cold exposure, distinct transcriptional profiles were observed between control (C) and cold-stressed (D) groups. Principal component analysis (PCA) of significantly different genes demonstrated clear separation of samples into 10 clusters (Figure 1A), signifying pronounced differences between the groups and attesting to the high repeatability and reliability of the data. Volcano plots (Figure 1B) identified 1588, 1457, 2121, 1576, and 1455 DEGs for between C-OB vs. D-OB, C-FB vs. D-FB, C-MB vs. D-MB, C-CB vs. D-CB, and C-SC vs. D-SC, respectively. Notably, the MB region exhibited the highest number of DEGs, suggesting that it underwent the most significant alterations in response to cold stress.

### 3.3. GO Enrichment of DEGs in Brain Tissue

To elucidate the biological roles of DEGs, all identified DEGs were functionally annotated using the GO database. Statistical significance was assessed by the Fisher method, with adjusted *p*-values < 0.05 considered indicative of significant enrichment. All DEGs were categorized into molecular function (MF), cellular component (CC), and biological process (BP) ontologies; full results are displayed in Figure S1.

**Figure 2:**
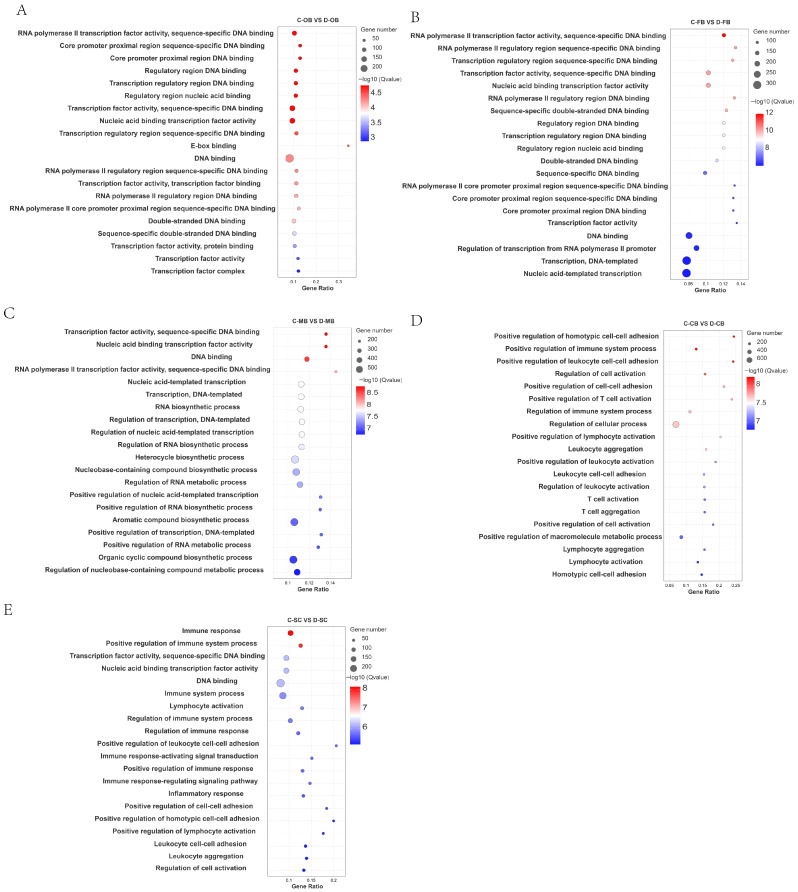
GO enrichment of DEGs in different brain regions of *L. longirostris* after cold stress. (**A**–**E**) GO enrichment analysis of DEGs for C-OB vs. D-OB, C-FB vs. D-FB, C-MB vs. D-MB, C-CB vs. D-CB, and C-SC vs. D-SC, respectively. The vertical axis represents the name of the pathway, and the horizontal axis Rich factor represents the sample number/background number ratio. The size and color of the dots represent the number of genes and the adjusted *p* for each pathway, respectively.

Analysis of the top 20 enriched GO terms (Figure 2) revealed distinct functional patterns. In the comparisons C-OB vs. D-OB, C-FB vs. D-FB, and C-MB vs. D-MB (Figure 2A–C), enriched functions predominantly involved nucleic acid metabolism, including DNA binding, RNA biosynthesis, transcription factor activity, and regulation of DNA-templated transcription. This pattern implies that cold stress impacts transcriptional regulation. Conversely, the enriched GO functions between C-CB vs. D-CB and C-SC vs. D-SC (Figure 2D,E) were primarily associated with immune-related processes, such as immune response, positive regulation of T cell activation, and leukocyte aggregation, suggesting that cold stress induces an immune response within brain tissue.

Collectively, these findings demonstrate region-specific functional specialization within the brain during cold adaptation.

### 3.4. KEGG Enrichment of DEGs in Brain Tissue

DEGs were further mapped to the KEGG database for pathway enrichment analysis (Figure S2). Pathways with adjusted *p* < 0.05 (Fisher’s test) were deemed significantly enriched.

**Figure 3:**
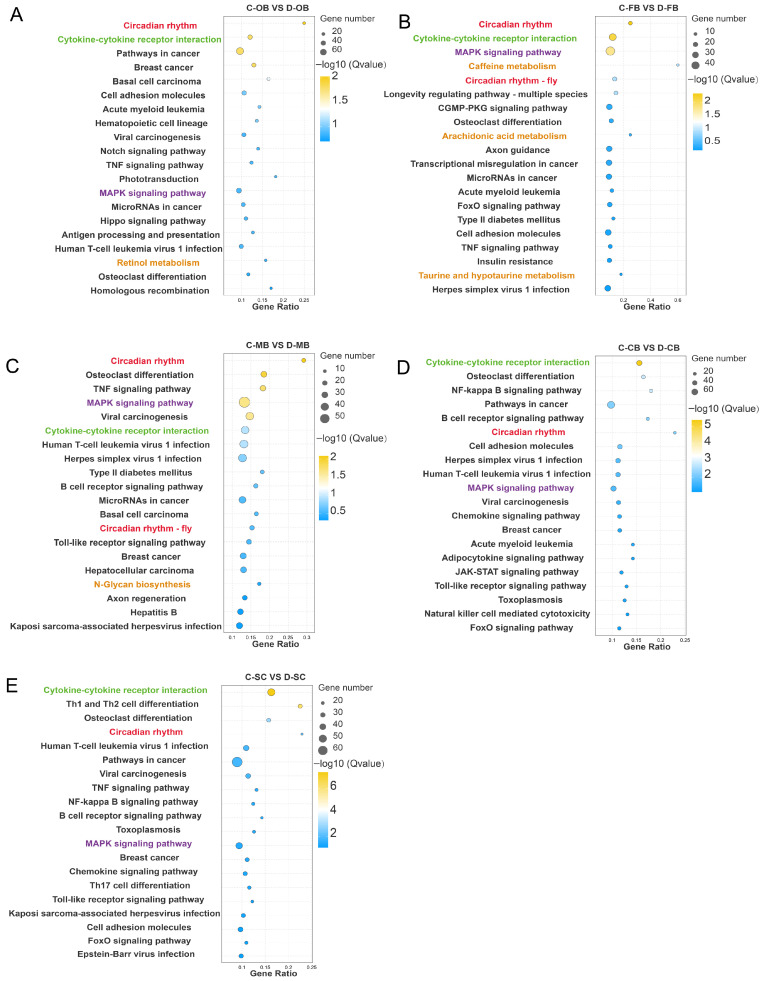
KEGG enrichment of DEGs in different brain regions of *L. longirostris* after cold stress. (**A**–**E**) KEGG enrichment analysis of DEGs for C-OB vs. D-OB, C-FB vs. D-FB, C-MB vs. D-MB, C-CB vs. D-CB, and C-SC vs. D-SC, respectively. The vertical axis represents the name of the pathway, and the horizontal axis Rich factor represents the sample number/background number ratio. The size and color of the dots represent the number of genes and the adjusted *p* for each pathway, respectively.

According to the top 20 enrichment pathways identified, a substantial number of metabolic processes, including retinol metabolism, taurine and hypotaurine metabolism, and arachidonic acid metabolism were enriched in the OB, FB, and MB regions (Figure 3A–C), while no such pathways were enriched in the CB and SC regions (Figure 3D,E). This regional divergence aligns with GO results, reinforcing differential cold responses across brain compartments. Notably, the responses of the OB, FB, and MB regions exhibited greater similarity, whereas the responses of the CB and SC demonstrated a closer resemblance. Moreover, circadian rhythm, MAPK signaling pathway, and Cytokine-cytokine receptor interaction processes were extensively enriched in all five brain regions (Figure 3A–E), indicating that the biological clock, stress signal sensing, intracellular transduction and immune response are essential for the adaptation and survival of *L. longirostris* under cold stress conditions.

### 3.5. Effects of Cold Stress on Brain Immunity and Rhythm

To dissect the roles of cytokine-cytokine receptor interaction, circadian rhythm, and MAPK signaling in cold adaptation, we performed GSEA and selected the key genes within these three biological processes respectively for cluster analysis.

**Figure 4:**
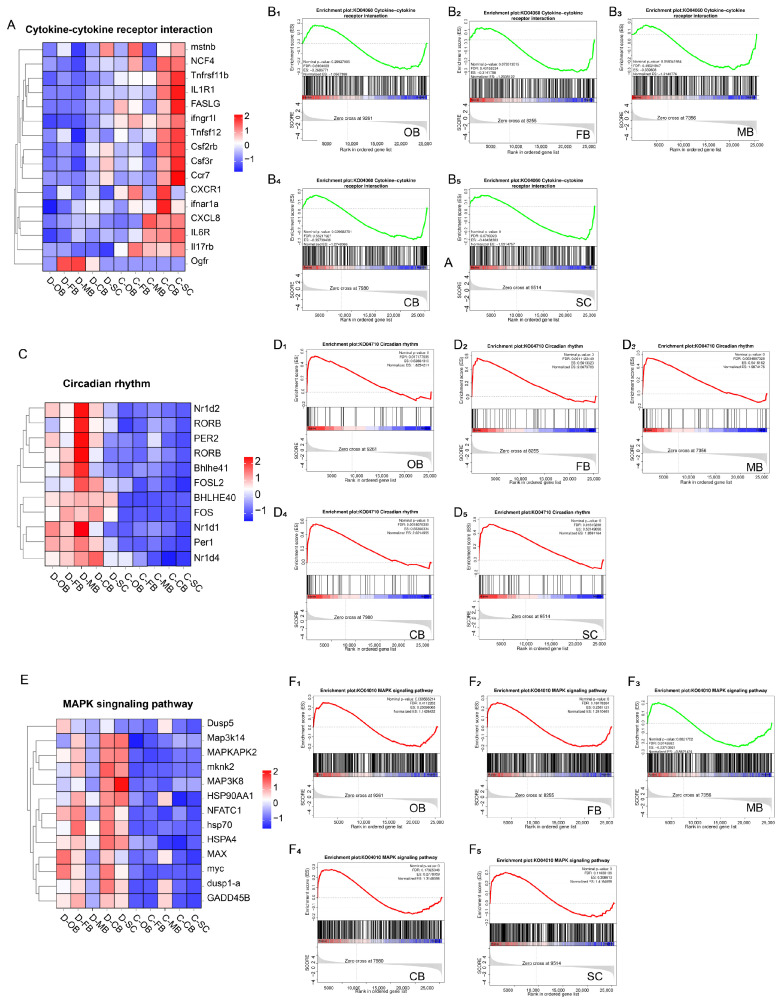
Effects of cold stress on the key biological pathways in brain of *L. longirostris.* (**A**) Hierarchical clustering analysis of key genes involved in the cytokine-cytokine receptor interaction pathway. (**B1**–**B5**) GSEA analysis related to cytokine-cytokine receptor interaction in five brain regions, respectively. (**C**) Hierarchical clustering analysis of key genes involved in the circadian rhythm pathway. (**D1**–**D5**) GSEA analysis related to circadian rhythm pathway in five brain regions, respectively. (**E**) Hierarchical clustering analysis of key genes involved in the MAPK signaling pathway. (**F1**–**F5**) GSEA analysis related to MAPK signaling pathway in five brain regions, respectively.

GSEA revealed significant downregulation of cytokine-cytokine receptor interactions across all brain regions in group D (Figure 4A,B), suggesting cold stress suppresses inflammatory defense and cellular differentiation. Conversely, circadian rhythm genes showed marked upregulation in group D (Figure 4C,D), most prominently in MB, implicating this region as a hub for biological clock reorganization. MAPK signaling displayed complex regulation: transcript levels of key genes increased in OB, FB, CB, and SC but decreased in MB of cold-exposed fish (Figure 4E,F), indicating that the MB region might respond in a distinct manner compared to other brain regions subsequent to cold stress.

### 3.6. WGCNA Identifies Cold-Responsive Gene Modules Associated with CIRBP

**Figure 5:**
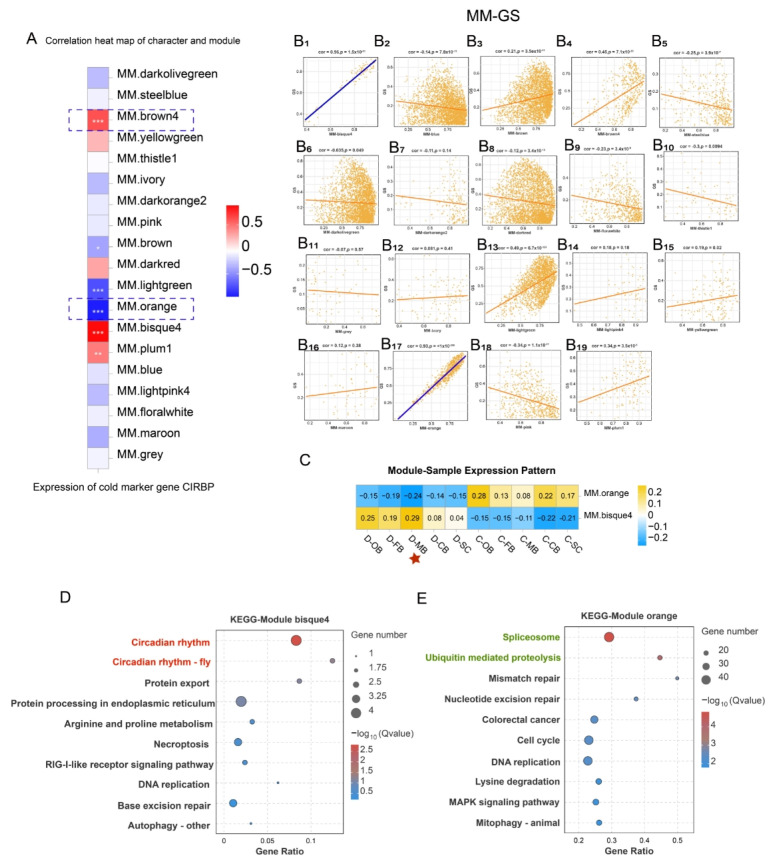
WGCNA after cold stress. (**A**) Correlation analysis of characters and modules. “*” indicates the significance of the correlation between modules and CIRBP expression, with more “*” representing a more significant correlation. (**B_1_**–**B_19_**) Module membership (MM) and Gene significance (GS) correlation analysis in 19 co-expressed gene modules. (**C**) Module -sample expression pattern of module bisque4 and module orange. (**D**) KEGG enrichment analysis of module bisque4. (**E**) KEGG enrichment analysis of module orange.

Weighted Gene Co-expression Network Analysis (WGCNA) identified 19 co-expressed gene modules (Figure S3) and their associations with cold response. The cold marker gene CIRBP belongs to a family of cold shock proteins that respond to cold shock [16,17] and serves as a universal marker for cold exposure in animals. We found that module brown4, module bisque4, and module plum1 exhibited a significantly positive correlation with CIRBP expression, whereas module brown, module light-green, and module orange displayed a significantly negative correlation with CIRBP expression (Figure 5A).

To probe into the correlation between module membership (MM) and CIRBP expression (Gene significance, GS) for each module, MM-GS correlation analysis was conducted on the 19 modules. The MM-GS correlation analysis highlighted bisque4 and orange as the modules most significantly associated with CIRBP expression (Figure 5B), designating them as key cold-responsive modules.

The expression patterns of genes in module bisque4 and module orange within each sample were visualized via module eigenvalues, and a heat map of the sample expression patterns was generated. The results demonstrated that the comprehensive expression levels of all genes in module bisque4 and module orange were the highest in the D-MB samples (Figure 5C), confirming MB’s pivotal role. Furthermore, KEGG enrichment of these modules identified circadian entrainment pathways in bisque4 module (Figure 5D), while orange module genes enriched for spliceosome and ubiquitin-mediated proteolysis (Figure 5E).

**Figure 6:**
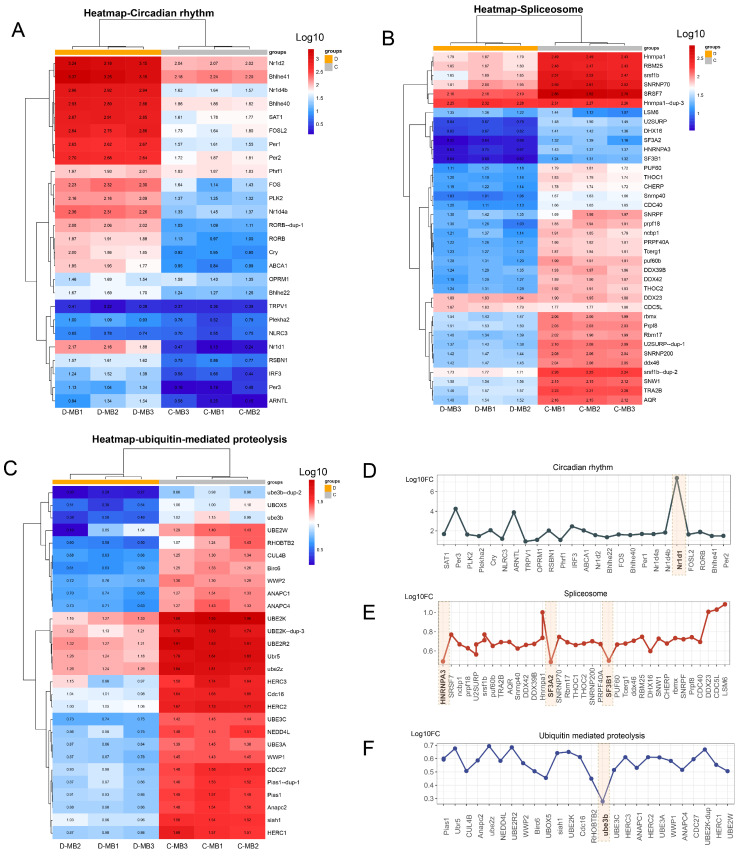
Analysis of key biological processes in MB region after cold stress. (**A**–**C**) Cluster analysis of all genes annotated to circadian rhythm in module bisque4, to spliceosome in module orange, and ubiquitin-mediated proteolysis in module orange, respectively. (**D**–**F**) Fold change of all genes annotated to circadian rhythm in module bisque4, to spliceosome in module orange, and ubiquitin-mediated proteolysis in Module orange, respectively.

To further investigate the alterations in circadian rhythm, spliceosome, and ubiquitin-mediated proteolysis within the MB region after cold stress, we performed cluster analysis on all genes annotated to these three pathways. The results revealed that compared with the control group, circadian rhythm-related genes showed global upregulation (Figure 6A), with Nr1d1 as the top-induced gene (Figure 6D), suggesting its role as a core clock regulator. Regarding the spliceosome, related genes demonstrated a significant down-regulation subsequent to cold stress (Figure 6B) with the most prominently down-regulated markers were identified as HNRNP A3, SF3A2, and SF3B1 (Figure 6E). Furthermore, ubiquitin-mediated proteolysis was also inhibited following cold stress (Figure 6C), with Ube3b as the most downregulated marker (Figure 6F).

## 4. Discussion

### 4.1. The Mesencephalon Plays an Important Role in the Life Activities of Fish

Most fish are poikilotherms, able to sense alterations in ambient temperature and adapt through physiological and behavioral regulation. The mesencephalon (MB) is usually the largest brain area of fish, and also the highest visual center, which plays a pivotal role in fish life activities [18]. Previous studies have noted that cold-sensitive neurons may exist in the MB, thereby playing a role in temperature signal transmission and regulation [19].

Transcriptomic profiling in this study identifies the mesencephalon (MB) as the predominant hub for cold stress response in *L. longirostris*, as MB has the largest number of DEGs among all brain regions and is most strongly associated with cold-responsive co-expression modules (bisque4 and orange). Therefore, we hypothesized that MB plays a core role in cold adaptation, integrating temperature signals via interactions among cold-sensitive, visual, and motor neurons, to coordinate adaptive physiological and behavioral responses.

However, through analysis, only a basic conclusion was drawn in this study: the MB may be the primary brain region that regulates the response to cold stress in *L. longirostris*. The specific molecular networks and critical cell types mediating MB-dependent cold adaptation in *L. longirostris* still need further investigation.

### 4.2. Cold Stress Affects the Biological Clock

Almost all eukaryotic cells possess a self-sustaining biological clock that combines endogenous biochemical, physiological, and behavioral rhythms with environmental changes [20]. This circadian system maintains tissue homeostasis amid environmental fluctuations [21] with rhythms modulated by light, nutrients, and temperature [22]. When the circadian system is disturbed, pathological consequences follow, inducing metabolic disorders and an increased risk of disease [23].

The effect of temperature on the circadian clock of fish has been well established. Even a minor temperature fluctuation of only 2 °C is capable of perturbing the amplitude of the circadian rhythm [24]. Zebrafish exhibit upregulation of core clock genes after cold stress (such as Nr1d isoforms and Per homologs) [25], concordant with Liu et al.’s report of cold-induced upregulation of *CIRBP* and circadian genes in *Puntius tetrazona*’s brain [7].

These findings resemble our results: circadian rhythm pathways were enriched across all brain regions, with significant upregulation of *Nr1d1*, *Nr1d2*, and *Nr1d4*. WGCNA further linked circadian entrainment to module bisque4. These results imply that circadian clocks are involved in cold adaptation. Moreover, since Nr1d proteins act as transcriptional silencers regulating circadian rhythms, lipid metabolism, and differentiation [26], their induction may drive broad transcriptional suppression during cold adaptation. In addition, Nr1d1 emerged as the most conspicuously upregulated circadian rhythm factor within the MB region, highlighting its potential as a master regulator in the overall cold adaptation mechanism.

### 4.3. Cold Stress Affects Immune Function and Inflammatory Response

The cerebellum (CB), modulating several regions of the hypothalamus, could be considered as an important participant in regulating psychoneuroendocrine immunology [27]. The spinal cord (SC) serves as a peripheral immune afferent hub, where the microglia participate in innate/adaptive immune responses by secreting pro-inflammatory factors and chemokines [28]. Therefore, both the CB and the SC have neuroimmune regulatory functions. This study revealed that CB and SC specifically enrich immunomodulatory pathways such as “positive regulation of T cell activation” and “leukocyte aggregation” under cold stress, suggesting their involvement in maintaining neuroinflammatory homeostasis through adaptive immune cell activity modulation.

MAPK signaling critically regulates immune, inflammatory, and stress responses. In the present study, the MAPK signaling pathway presented a brain region-specific heterogeneous activation pattern in response to cold stress: it was significantly activated in the CB and SC, which might enhance the cellular antifreeze capacity by inducing the expression of heat shock protein (HSP) and antifreeze protein (AFP), thereby facilitating adaptation to cold stress [29]. In contrast, marked suppression in the MB might mitigate the risk of inflammation exacerbated by MAPK hyperactivation [30]. This differential regulation indicates that the CB and SC maintain basic immune defense via moderate MAPK activation, while the MB avoids excessive inflammatory damage by inhibiting MAPK.

Cytokine-cytokine receptor interactions critically modulate inflammatory intensity/duration. For instance, the chemokine receptor CXCR3 regulates inflammatory intensity by recruiting Th1 cells to inflammatory sites via binding ligands (CXCL9/10/11). Meanwhile, the CXCR3-CXCL10 axis influences the duration of inflammation through Th1/Th2 balance modulation. Changes in its expression can disrupt the persistent pro-inflammatory balance and affect the infiltration of inflammatory cells and the release of cytokines [31]. In this study, the cytokine—cytokine receptor pathway was significantly downregulated across all brain regions post-cold stress, which was consistent with the downregulation trend of pro-inflammatory receptors such as IL-1R, IL-6R, and IL-17R. Such modulation may contribute to the prevention of excessive immune inflammatory response and duration, thereby safeguarding immune homeostasis.

In summary, the observed region-specific alterations in cerebral immune pathways following cold stress in this study are physiologically significant. The global suppression of cytokine-receptor interactions, concomitant with the downregulation of pro-inflammatory receptors, likely represents a protective mechanism to prevent excessive inflammatory injury. This precise immunomodulation is particularly crucial in hypothermic environments, where an excessive inflammatory response could exacerbate energy expenditure and induce tissue damage, whereas moderate suppression balances immune defense with energy conservation for survival. Notably, the specific inhibition of MAPK signaling within the MB region further mitigates the risk of pro-inflammatory signal transduction, demonstrating how distinct brain regions employ coordinated modulation to avoid systemic immune imbalance.

### 4.4. Cold Stress Affects the Gene Expression Process

Under low temperature, the reduced molecular thermal motion increases the probability of hydrogen bond formation between the bases within RNA molecules, leading to the complexity of the secondary structure of RNA and ultimately affecting the transcription, splicing, and translation processes [32]. Numerous genes have been identified as potential molecular markers for characterizing cold stress responses in diverse fish species, including cold-induced RNA-binding proteins (cirbp), ribonucleoproteins (snrpd3, prpf8), and translation factors (eif1axa, eief2a) [33]. Andrew et al. [34] observed enrichment in RNA splicing, ribosome biogenesis, and DNA binding in cold-stressed poikilotherms. Similarly, OB, FB, and MB exhibited GO term enrichment for nucleic acid synthesis/regulation (such as DNA binding, RNA biosynthesis) post-cold exposure, suggesting that cold stress exerts an impact on the regulation of gene expression in *L. longirostris*.

Selective splicing events are closely associated with the spliceosome [35], and there is accumulating evidence indicating that the expression of spliceosome-related genes plays a necessary role in adapting to various stress conditions [36]. In this study, the spliceosome was the most significantly enriched pathway within module orange, and cold stress suppressed the majority of U1/U2/U4-U6/U5-associated genes’ expression. Besides, critical splicing regulators such as HNRNP A3 (involved in spliceosome assembly and splice site selection), SF3A2, and SF3B1 (involved in stabilizing U2/branch site duplex) [37] were markedly downregulated after cold stress.

In light of the above results, we deduced that the synthesis, transcription, and translation in *L. longirostris* nucleic acid are disrupted under extreme cold conditions, which may potentially lead to a reduction in stress resistance at the physiological level. Moreover, the core responsive region was MB, with HNRNP A3, SF3A2, and SF3B1 as key biomarkers.

### 4.5. Effect of Cold Stress on Ubiquitination

Extreme temperatures can cause protein misfolding and produce cytotoxicity [38], and the accumulated misfolded proteins necessitate ubiquitin-mediated degradation via E1 (activating), E2 (conjugating), and E3 (ligating) enzymes [39].

Ubiquitin-mediated protein degradation is essential for fish to cope with acute cold stress. For instance, Liu et al. [7] found that, upon exposure to acute cold stress, ubiquitination-related genes were significantly expressed in temperate fish (such as *Cyprinus carpio*) and tropical fish (such as *Danio rerio*). Moreover, five genes associated with ubiquitination/deubiquitination were identified as cold-induced DEGs, including TULP4, WWP2, PPARD, CUL9, and USP4.

In this study, we also enriched ubiquitination-mediated proteolysis within module orange, suggesting that the brain response to cold stress is closely related to the process of ubiquitination. Interestingly, however, ubiquitin-mediated proteolysis genes were transcriptionally suppressed in MB. This inhibition likely disrupts protein homeostasis following acute cold stress.

### 4.6. Effect of Cold Stress on Nutrition Metabolism

Cold stress elicits comprehensive systemic metabolic reprogramming, involving multi-organ adjustments in nutrient utilization. As evidenced in murine models, cold exposure elevates plasma free fatty acids as primary substrates for TCA cycle energy generation [40]. Similarly, the impact of cold stress on nutritional metabolic pathways may also be directly related to the energy distribution strategies of fish. For instance, in *Chanos chanos*, acute cold stress initially increases glucose mobilization, while prolonged exposure shifts metabolic reliance toward lipid catabolism to sustain essential functions [41].

In this study, significant enrichment of retinol metabolism, taurine and hypotaurine metabolism, and arachidonic acid metabolism pathways in the OB, FB, and MB regions reflects adaptive adjustments in energy utilization and neuroprotection during cold stress. The retinol metabolism enrichment implies vitamin A derivatives’ involvement in neural protection and visual adaptation critical for hypothermic foraging [42]. The enrichment of taurine metabolism may enhance the brain’s tolerance to low-temperature stress by regulating antioxidant effects [43], while the arachidonic acid metabolism activation maintains neuronal membrane fluidity via regulated proportion of unsaturated fatty acids [44].

Collectively, these metabolic changes reflect a comprehensive adaptive strategy of brain tissue at low temperatures: by optimizing energy allocation, strengthening neuroprotection, and preserving membrane integrity, thereby maintaining the homeostasis and survival ability of the nervous system.

While our study provides valuable insights into the differential expression patterns in brain regions of group C and group D, it is important to acknowledge that the use of three biological replicates per brain region represents a minimal sample size for transcriptome analysis. This limitation may affect the statistical power of our analyses and the reproducibility of the differential expression results. Specifically, a smaller sample size can increase the likelihood of false positives and false negatives, potentially leading to less robust conclusions. Future studies with larger sample sizes are needed to validate our findings and enhance the reliability of the differential expression results.

## 5. Conclusions

This study revealed the regional heterogeneity in the cold stress response among different brain regions (OB, FB, MB, CB, and SC) of *L. longirostris*. Specifically, the OB, FB, and MB are primarily involved in metabolic regulation and transcriptional control, whereas the CB and SC are mainly participated in immune defense. Of particular significance, the MB has been identified as the core regulatory hub in *L. longirostris* for cold stress response. It coordinates the overall adaptive reactions through regulating circadian clock reorganization, immune function modulation, and gene expression control (including the inhibition of spliceosome and ubiquitination pathways). However, the specific molecular network of MB in regulating cold stress has not yet been elucidated, and the physiological functions of spliceosome inhibition and circadian gene regulation lack experimental verification. In the future, it is necessary to locate the key cell populations of MB through single-cell sequencing, verify the pathway function through gene editing (such as knockdown of *Nr1d1*), and cultivate strains with stable spliceosome function to facilitate cold-resistant molecular breeding.

## Data Availability

The RNA-seq data is available in the National Center of Biotechnology Information (NCBI) database via accession number PRJNA1208664.

## Supplementary Materials

The following supporting information can be downloaded at: https://www.mdpi.com/article/10.3390/ani15142107/s1, Figure S1: The annotation of all differential genes in the GO database; Figure S2: The annotation of all differential genes in the KEGG database; Figure S3: Power value curve and cluster module partitioning of WGCNA; Table S1: Summary table of base quality for sequencing data; Table S2: The outcomes of mapping comparison with reference genomes.
