# Supplementary figures and images for: RNA-Seq Revealed the Effects of Cold Stress on Different Brain Regions of Leiocassis longirostris
Source: Animals (Basel). 2025 Jul 16;15(14):2107. doi: 10.3390/ani15142107 (PMC12291733; doi:10.3390/ani15142107)

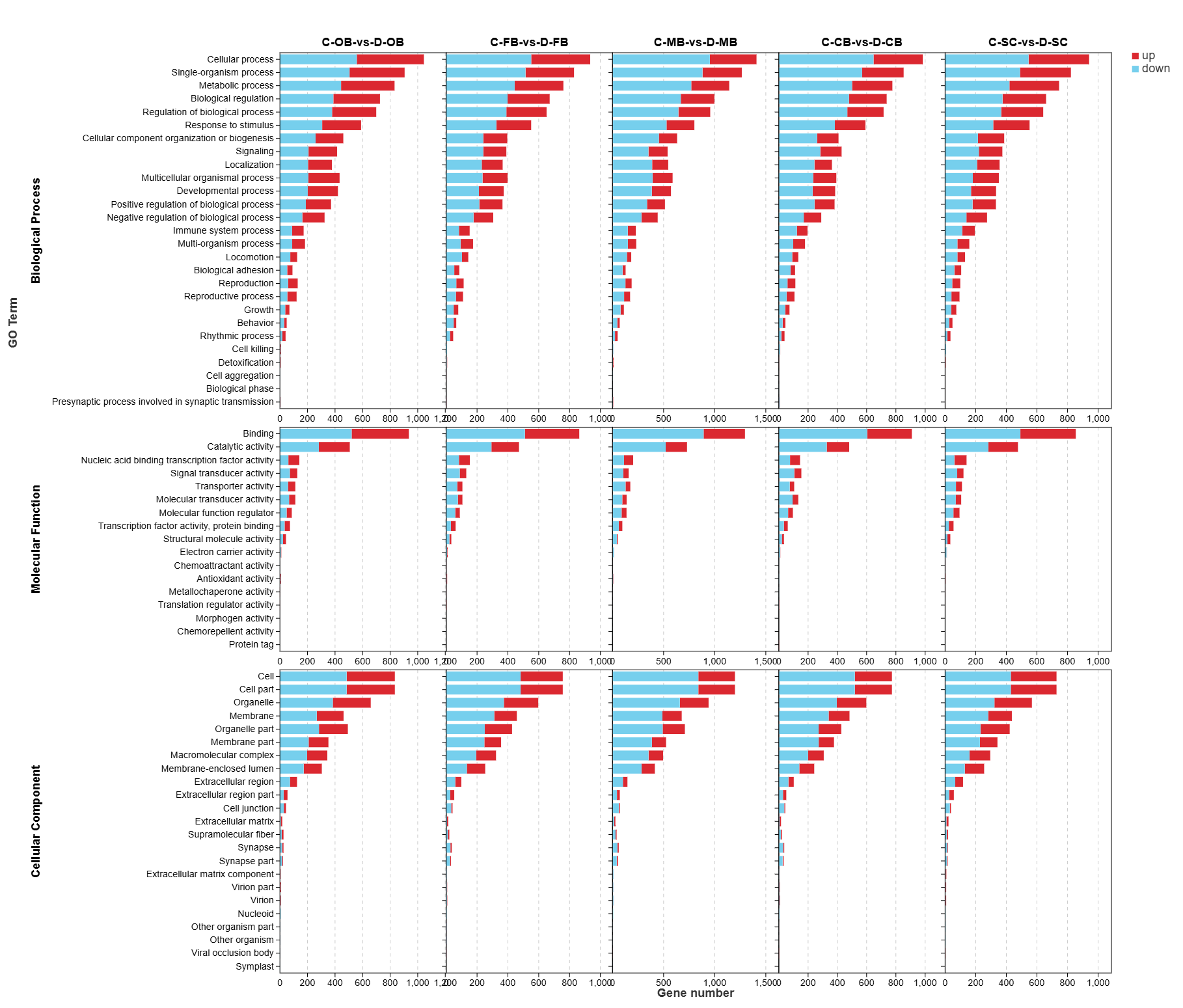

Supplement: Supplementary file 1 [file animals-15-02107-s001.zip › Supplementary File/FigS1.png]

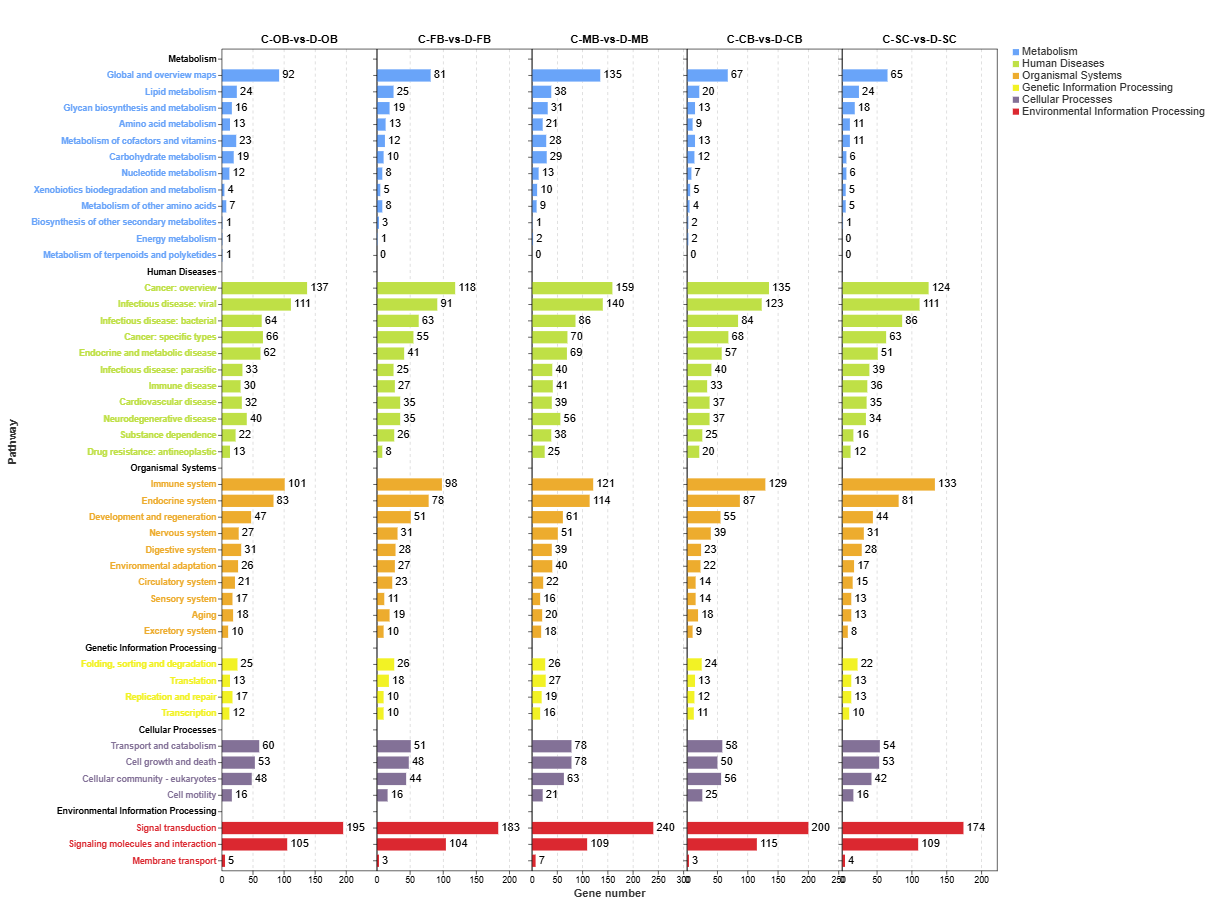

Supplement: Supplementary file 1 [file animals-15-02107-s001.zip › Supplementary File/FigS2.png]

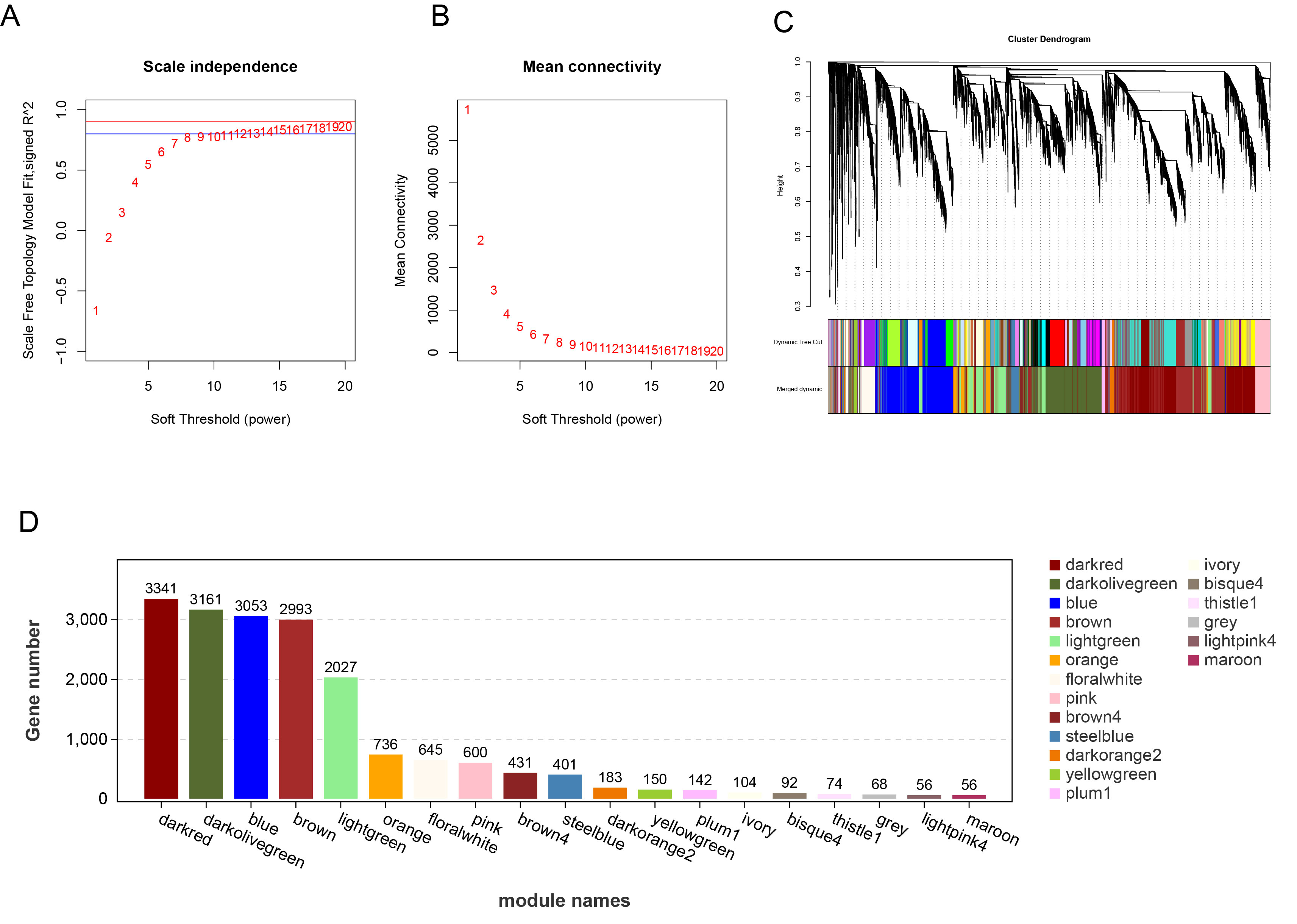

Supplement: Supplementary file 1 [file animals-15-02107-s001.zip › Supplementary File/FigS3.png]
